# Regulation of transient receptor potential ankyrin 1 by traditional Chinese medicine drugs and their active ingredients

**DOI:** 10.3389/fphar.2025.1604765

**Published:** 2025-06-06

**Authors:** Yunlong Fang, Chunxin Lei, Zihan Wang, Longxiao Liu, Yanyu Chen, Feng Chen, Jingxuan Li, Zhikun Tu, Qingwen Tao, Yuan Xu

**Affiliations:** ^1^ National Center for Integrative Medicine, Department of TCM Rheumatism, China-Japan Friendship Hospital, Beijing, China; ^2^ Graduate School, Beijing University of Chinese Medicine, Beijing, China; ^3^ Graduate School, Yunnan University of Chinese Medicine, Kunming, Yunnan, China; ^4^ Graduate School, Shanxi University of Chinese Medicine, Taiyuan, Shanxi, China

**Keywords:** TRPA1, traditional Chinese medicine, Chinese herbal medicinal properties, natural compounds, pain, hyperalgesia, inflammatory disease

## Abstract

Transient receptor potential ankyrin 1 (TRPA1) has attracted substantial attention for its role as a thermal receptor and regulator of temperature and pain sensing. Notably, Traditional Chinese Medicine (TCM) has demonstrated pharmacological effects through regulation of TRPA1. In this review, we summarize the regulatory effects of TCM drugs and their active ingredients on TRPA1. A total of 31 TCM drugs were identified and categorized into three groups based on their function. Despite the progress in understanding the regulatory effects of TCM drugs and their constituents on TRPA1, further studies are needed to elucidate the molecular mechanisms involved. This review provides a molecular basis for the pharmacological effects of TCM drugs on TRPA1 regulation and offers evidence to support future research in this area.

## 1 Introduction

**FIGURE 1:**
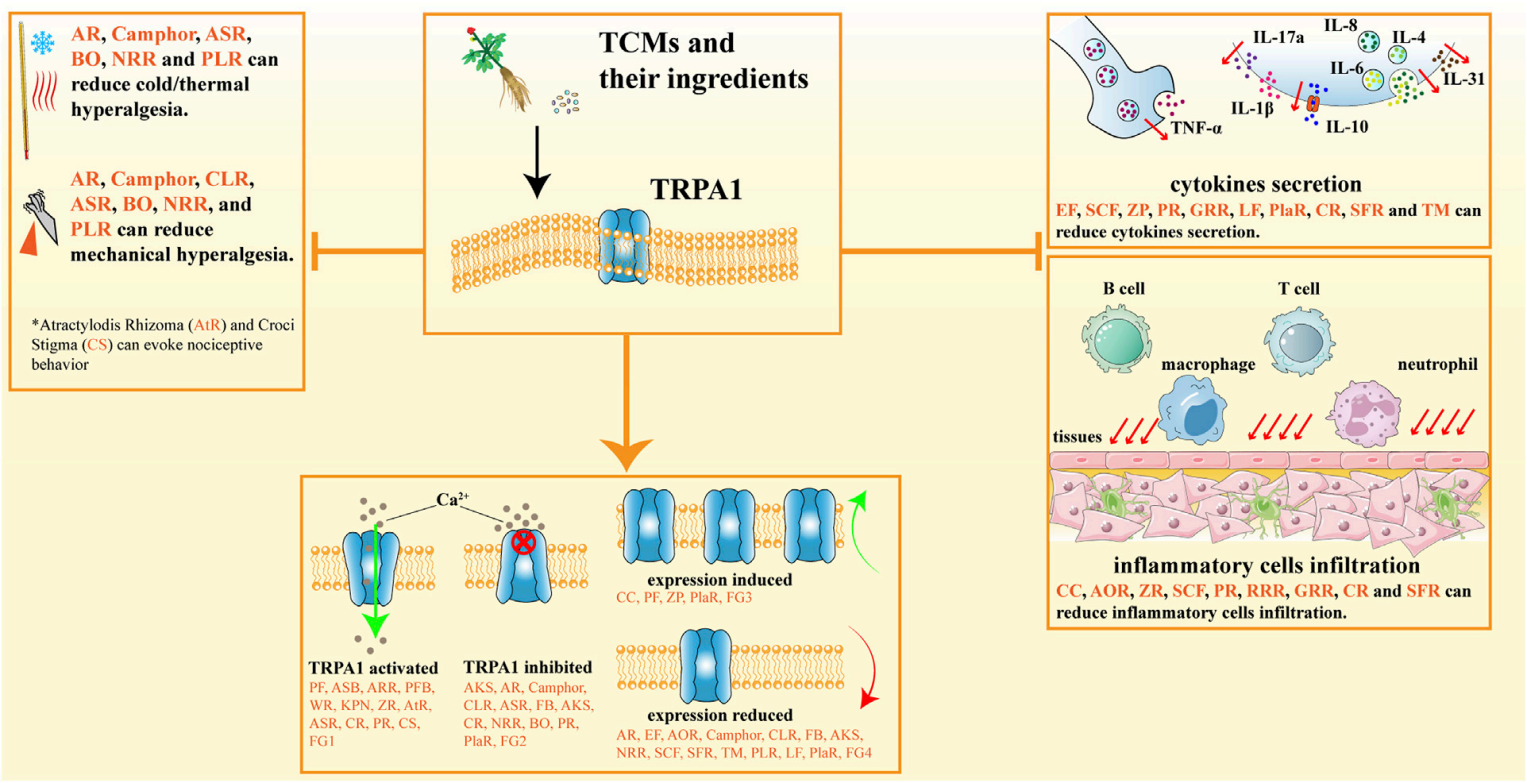
Regulation of TRPA1 by TCM drugs and their ingredients. Through the regulation of TRPA1, TCM drugs and their ingredients can alleviate cold/thermal hyperalgesia, mechanical hyperalgesia, and reduce cytokine secretion and inflammatory cell infiltration. AKS: Alpiniae Katsumadai Semen; AOR: Alpiniae Officinarum Rhizoma; AR: Aconiti Radix; ARR: Asari Radix et Rhizoma; ASB: Allii Sativi Bulbus; ASR: Angelicae Sinensis Radix; AtR: Atractylodis Rhizoma; BO: Borneol; CC: Cinnamomi Cortex; CLR: Curcumae Longae Rhizoma; CR: Chuanxiong Rhizoma; CS: Croci Stigma; EF: Evodiae Fructus; FB: Faeces Bombycis; GRR: Glycyrrhizae Radix et Rhizoma; KPN: Kalopanax pictus Nakai; LF: Lycii Fructus; NRR: Notopterygii Radix et Rhizoma; PF: Piperis Fructus; PFB: Perillae Fructus/Perillae Folium; PlaR: Platycodi Radix; PLR: Puerariae Lobatae Radix; RR:Paeoniae Radix; RRR: Rhei Radix et Rhizoma; SCF: Schisandrae Chinensis Fructus; SFR:Sophorae Flavescentis Radix; TM: Tetrapanacis Medulla; WR: Waldheimia glabra (Decne.) Regel; ZP: Zanthoxyli Pericarpium; ZR: Zingiberis Rhizoma; FG1 (Formula group 1): Daikenchuto(Da-Jian-Zhong-Tang), Maobushisaishinto (Mahuang Fuzi Xixin Decoction), Tokishakuyakusan (Danggui Shaoyao San); FG2 (Formula group 2): Danggui Sini Decoction, Goshajinkigan (Jisheng Shenqi Pills), Banhasasim-tang (Banxia Xiexin Decoction), Shugan Decoction, Wu-Tou Decoction, San’ao Decoction; FG3 (Formula group 3): Daikenchuto(Da-Jian-Zhong-Tang); FG4 (Formula group 4): Danggui Sini Decoction, Zhenxin Anshen Formula, Houpo-Mahuang Decoction, Yanghe Decoction Qingpeng Ointment, Sanse Powder, Wu-Tou Decoction, San’ao Decoction, Chi-Huang Solution.

Traditional Chinese Medicine (TCM) is a widely used system of medicine in China, with a long history of effectiveness in addressing numerous complex diseases and major epidemics. TCM formulas are typically composed of multiple herbs, each containing various active ingredients targeting specific protein sites, thus making them highly valuable for research and potential sources for new drug development (Tu, 2011). Clinical practice in TCM reflects a unique theory based on the Yin-Yang doctrine and describes disease conditions through the two basic patterns of cold and heat. TCM classifies herbs based on four qi (hot, warm, cold, and cool), with hot and warm herbs alleviating cold symptoms such as hypersensitivity to cold and wind, and cold and cool herbs alleviating heat symptoms such as skin redness, swelling and skin burning sensation. Transient receptor potential (TRP) ion channels are recognized as sensors of coldness and warmth (Paricio-Montesinos et al., 2020), of which transient receptor potential ankyrin 1 (TRPA1) has been identified as a cold-activated channel (Talavera et al., 2020; Yan et al., 2022) that plays an important role in regulating inflammation and pain (Souza Monteiro de Araujo et al., 2020; Balestrini et al., 2021) (Figure 1).

At present, reviews that focus on the regulation of TRPA1 by TCM are lacking. This review summarizes the studies on TCM drugs that influence the functioning and proliferation of TRPA1, thereby offering a comprehensive overview and fostering a deeper understanding of the mechanisms underlying the pharmacological effects of TCM drugs. This essay will also serve as a reference and outline potential avenues for future research on novel drug development and modern application of TCM.

## 2 TRPA1

### 2.1 Structure

The *TRPA1* gene, also known as *ANKTM1*, *FEPS*, *FEPS1*, *p120*, is a protein-coding gene. In humans, the *TRPA1* gene is located on chromosome 8, band q21.11, and consists of 68,761 base pairs and 29 exons according to the updated data on NCBI. Notably, the *TRPA1* gene is not limited to humans (hTRPA1) but is also found in numerous other species, including the house mouse (mTRPA1), fruit flies, dogs, and chickens.

Cryo-electron microscopic analysis has revealed that functional TRPA1 channels assemble as homotetramers through domain-swap interactions (Cvetkov et al., 2011). Each subunit consists of six transmembrane α-helices (S1-S6) flanking a re-entrant pore loop between S5 and S6, which collectively form a central cavity containing two distinct constrictions. These structural bottlenecks serve as putative gates that regulate calcium ion permeation. Notably, dual-pore helices positioned at the channel entrance exhibit selective anion exclusion through electrostatic-repulsion mechanisms (Paulsen et al., 2015).

**FIGURE 2:**
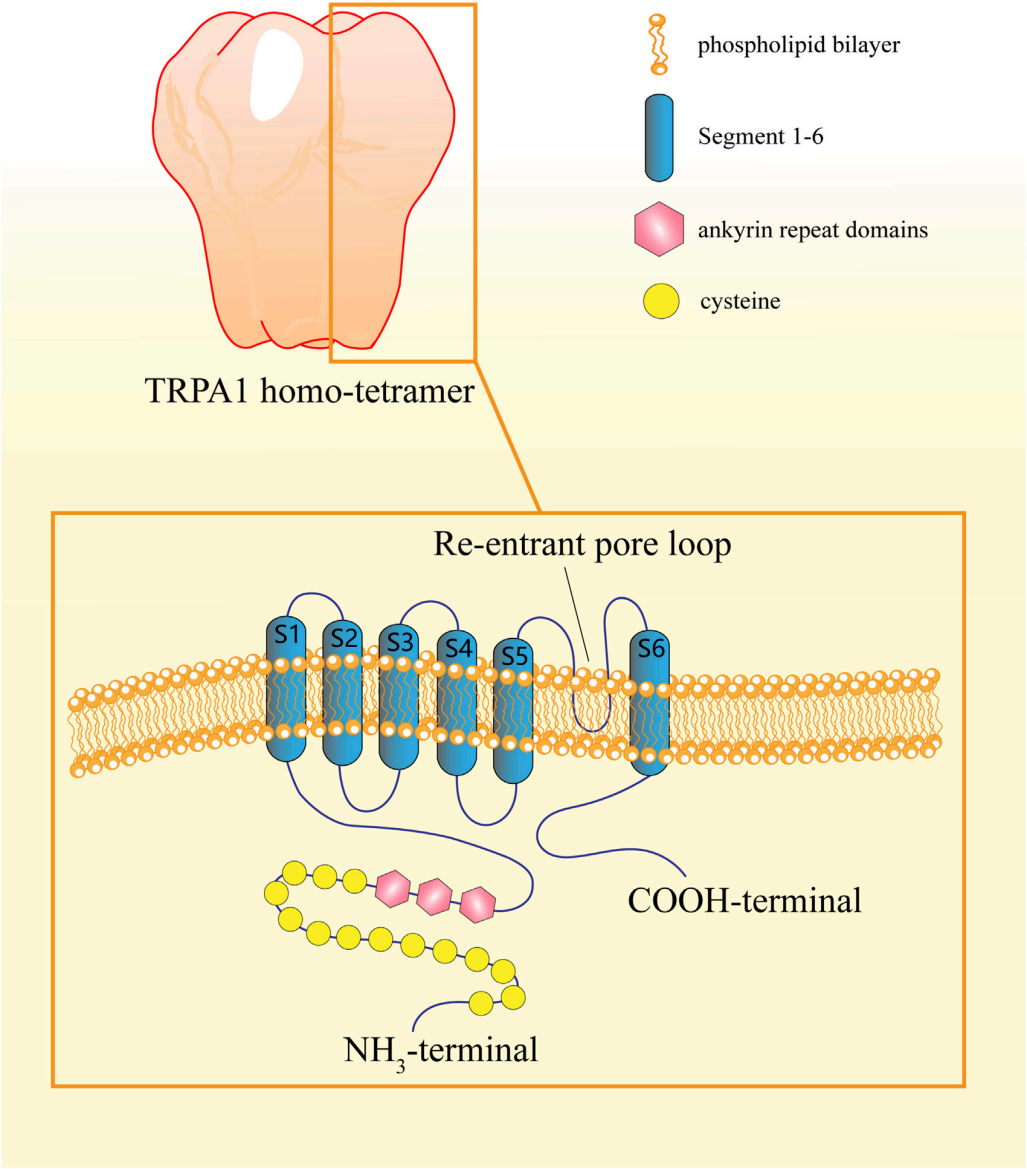
Structure of the TRPA1 protein. The TRPA1 protein, which assembles in a homo-tetramer, is composed of six segments and has a re-entrant pore loop between segments five and 6. The NH_2_-terminal portion possesses between 14 and 18 ankyrin repeat domains (ARDs), which control protein insertion and stability in the plasma membrane, and the COOH-terminal portion contains an α-helical TRP-like domain responsible for allosteric regulation of TRPA1.

The intracellular domains constitute approximately 80% of TRPA1’s molecular mass (Talavera et al., 2020). The extensive NH_2_ terminus contains 14–18 ankyrin repeat domains (ARDs) that orchestrate membrane insertion, structural stability, and protein–protein interactions (Gaudet, 2008; Nilius et al., 2011). A unique β-hairpin-loop connector, which consists of tandem α-helices and a pre-S1 helix that structurally bridges the transmembrane core with the ARD cluster, precedes S1 (KÁDkovÁ et al., 2017). This N-terminal region also harbors critical cysteine and lysine residues essential for agonist-binding specificity (Souza Monteiro de Araujo et al., 2020). The other terminal of the protein is the COOH-terminal portion, which demonstrates evolutionary conservation with other TRP channels, featuring a characteristic TRP-box motif immediately following S6. This α-helical structure engages in allosteric regulation through spatial proximity to both the pre-S1 helix and distal structural elements (Paulsen et al., 2015). Furthermore, TRPA1 possesses a putative EF-hand motif, a structural hallmark of calcium-binding proteins that may mediate its calcium sensitivity (Zygmunt and Högestätt, 2014) (Figure 2).

### 2.2 Functions and association with disease

As a nonselective cation channel, TRPA1 demonstrates permeability to monovalent (Na^+^, K^+^) and divalent (Ca^2+^) ions. It also shows notably higher calcium flux than other TRP family members (Zygmunt and Högestätt, 2014), a functional characteristic consistent with its distinctive structural features. This polymodal channel integrates diverse stimuli, including cold temperature (Kashio and Tominaga, 2022), osmotic pressure (Yao et al., 2023), and diverse electrophilic irritants ranging from environmental toxicants to endogenous algogenic lipids (Zhao et al., 2020). TRPA1 is widely distributed, appearing in the skin, airway, gastrointestinal tract, urinary system, vasculature, heart, and other locations (Earley, 2012; Yu et al., 2016; Wang M. et al., 2019; Maglie et al., 2021; Wu C. K. et al., 2021). However, its functional predominance is observed in nociceptive neurons. Specifically, TRPA1 shows preferential localization in peptidergic C-fibers (≈25% of total expression) and thinly myelinated Aδ-fibers within the peripheral nervous system (PNS), where it regulates the release of neuropeptides implicated in neurogenic inflammation, such as substance P and calcitonin gene-related peptide (CGRP) (Choi and Di Nardo, 2018). While central nervous system (CNS) expression of TRPA1 has been documented (Morelli et al., 2013; Kheradpezhouh et al., 2017), the central regulatory mechanisms are less clear than the well-characterized PNS functions.

**FIGURE 3:**
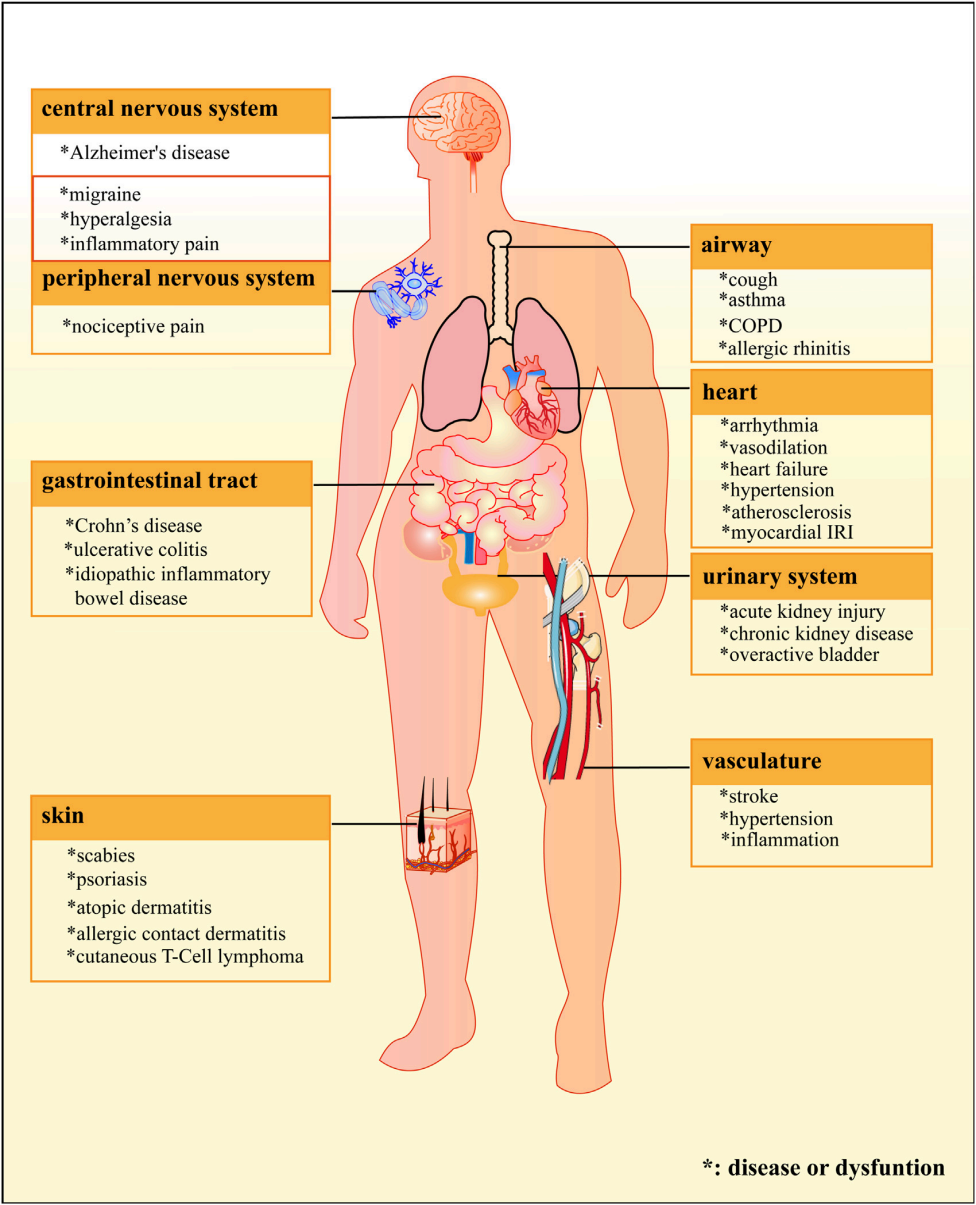
Locations of the TRPA1 protein and related diseases. The TPRA1 protein is widely distributed in different systems of human’s body, and is involved in pathological processes of numerous diseases.

The TRPA1 protein is widely expressed and can be regulated by various modulators. Therefore, it plays an important role in numerous physiological and pathological processes and is associated with a wide range of diseases, especially neurological disease, sensory-related disorders, and inflammation disease. The channel’s pathophysiological significance extends across multiple organ systems (Figure 3), serving as a crucial regulator in neuropathic pain (Kuruvilla et al., 2019), migraine (Benemei et al., 2013), itch (Moore et al., 2017), Alzheimer’s disease (Paumier et al., 2022), and some cardiovascular, gastrointestinal, respiratory, and urogenital diseases (Talavera et al., 2020). Recent studies have also reported that TRPA1 is involved in rheumatoid arthritis (Galindo et al., 2018), pancreatitis (Schwartz et al., 2013), diabetes, and obesity (Zsombok and Derbenev, 2016; Adhya and Sharma, 2019). Consequently, research and development of TRPA1 modulators holds great importance, given the large number of diseases related to TRPA1 and the anticipation that this number will continue to grow.

## 3 Studies on the drugs and their components in regulating pain by modulating TRPA1

Pain is a common symptom involved in various diseases and has been categorized under different classification schemes. Pain normally begins with noxious stimuli in the peripheral nerves, and is transmitted through the dorsal horn to the CNS (Lee and Neumeister, 2020). Numerous studies have shown that TRPA1 is located in the CNS, thus making it an important factor in the pathological process of pain (Souza Monteiro de Araujo et al., 2020). Moreover, TCM drugs have been shown to regulate pain through modulation of the production and function of TRPA1.

### 3.1 Aconiti Radix

Aconiti Radix (AR), which is derived from the dried root of *Aconitum carmichaelii* Debeaux (commonly referred to as Aconite, family: Ranunculaceae), is a traditional Chinese medicinal herb predominantly cultivated in southwest China’s mountainous regions (elevation, 1800–2000 m) (He G. et al., 2023). The principal bioactive diterpenoid alkaloid of AR, aconitine, mediates its therapeutic effects through multimodal pharmacological activities, including anticancer, antiviral, anti-inflammatory, and analgesic properties (Gao et al., 2022). Using calcium imaging, Jin et al. (2023) demonstrated direct TRPA1 channel blockade by aconitine (Maximal inhibition at 7 μM) and its capacity to downregulate TRPA1 expression in dorsal root ganglion (DRG) neurons of cancer-induced bone pain (CIBP) mice models. Notably, both aconitine and AR extract significantly ameliorated CIBP-associated cold/mechanical allodynia, indicating TRPA1-dependent analgesic mechanisms.

This pharmacological profile extends to classical AR-containing formulations. Wu-Tou Decoction, a canonical prescription for rheumatoid/osteoarthritis management (Zhou et al., 2020; Wu D. et al., 2021), was shown to mitigate multimodal hypersensitivity (mechanical, thermal, cold) in complete Freund’s adjuvant (CFA)-induced arthritis models (Wang et al., 2015). Mechanistically, the decoction suppressed TRPA1 channel activity while reducing its protein expression, highlighting conserved TRPA1 modulation across different AR-based therapeutic preparations.

### 3.2 Camphor

Camphor, a bicyclic monoterpenoid predominantly isolated from *Cinnamomum* spp. (family: Lauraceae), demonstrates broad-spectrum bioactivities encompassing insecticidal, antimicrobial, and antitussive effects, alongside its established role as a transdermal permeation enhancer (Chen W. et al., 2013). Historically rooted in TCM, this phytochemical has been empirically employed in managing stroke, dermatopathies, and gastrointestinal disorders. Modern pharmacological investigations by Li et al. (2023b) have revealed camphor’s potent suppression of TRPA1-mediated nociception: it was shown to attenuate allyl isothiocyanate (AITC)-evoked pain behaviors and ameliorate mechanical hypersensitivity in multiple TRPA1-dependent neuropathic pain models. These behavioral findings, corroborated by Xu et al. (2005), strongly implicate TRPA1 channel blockade as a primary mechanism underlying the analgesic effects of camphor.

Intriguingly, structural optimization of camphor derivatives revealed enhanced TRPA1 antagonism. Through comparative calcium imaging and voltage-clamp analyses, Takaishi et al. (2013) identified borneol and fenchyl alcohol analogs as superior human TRPA1 (hTRPA1) inhibitors, demonstrating 3- to 5-fold lower half-maximal inhibitory concentration (IC_50_) values and near-complete channel occlusion at equimolar concentrations *versus* camphor (IC_50_ = 1.26 ± 0.32 mM). This structure-activity relationship indicates the critical pharmacophore requirements for optimal TRPA1 interaction, which likely involve hydroxyl positioning and hydrocarbon skeleton rigidity. The authors consequently proposed prioritizing the development of these analogs as next-generation TRPA1-targeted therapeutics.

### 3.3 Atractylodis Rhizoma

Atractylodis Rhizoma (AtR), derived from the dried rhizomes of *Atractylodes lancea* (Thunb.) DC. and *Atractylodes chinensis* (DC.) Koidz (family: Asteraceae), has been a cornerstone of East Asian traditional medicine for managing gastrointestinal dysmotility, visceral hypersensitivity, night blindness and rheumatic disorders (Wei et al., 2017). Pharmacological studies have identified multimodal therapeutic properties in AtR extracts, including anticancer, anti-obesity, and anti-inflammatory activities (Koonrungsesomboon et al., 2014). Mechanistic investigations by Kanda et al. (2021) revealed that atractylodin (AtD), a principal sesquiterpenoid constituent of AtR, is a long-acting TRPA1 agonist. Using heterologous-expression systems, they demonstrated sustained Ca^2+^ influx in hTRPA1-HEK293 cells upon AtD application (EC_50_ = 0.913 μM), an effect that was entirely abolished in TRPA1-null DRG neurons. Notably, intraplantar AtD administration induced prolonged and moderate nociceptive behaviors in comparison with injection of AITC, establishing AtD as a TRPA1 agonist that possesses a long-lasting activation effect in mice and rats. In addition to AtD, other formulas containing AtR, such as Tokishakuyakusan (Danggui Shaoyao Decoction in Kampo medicine), have also been investigated. Shimizu et al. (2022) studied Tokishakuyakusan and found that the formula could restore thermoregulatory homeostasis and normalize blood flow after cold water immersion, probably through regulation of TRPA1. Then, they tested TRPA1 activation by some ingredients of AtR and identified atractylodin carboxylic acid as a potent agonist (EC_50_ = 4.4 μM).

### 3.4 Curcumae Longae Rhizoma

Curcumae Longae Rhizoma (CLR), also known as Tumeric, is the dried rhizome of *Curcuma longa* L. (family: Zingiberaceae), and has been traditionally employed to manage diverse pathologies spanning gastrointestinal, cardiovascular (e.g., heart pain), respiratory (e.g., runny nose, sinusitis, asthma), and hepatic disorders (Zhang and Kitts, 2021). The lipophilic polyphenolic constituent curcumin, which accounts for 2%–5% of CLR’s dry mass (Kocaadam and Şanlier, 2015), exhibits multi-channel-modulatory properties. Using high-throughput screening of thermosensitive TRP channels and taste receptors, Peng et al. (2022) identified curcumin-mediated inhibition of transient receptor potential vanilloid 1/2/3 (TRPV1/2/3) and TRPA1 (TRPA1 IC_50_ = 4.37 μM), while demethoxycurcumin (DMC) was shown to suppress TRPV3/TRPA1/TRPM8 activity in HEK293 overexpression models (TRPA1 IC_50_ = 7.35 μM).

Pharmacological exploration has also extended to synthetic derivatives. In streptozotocin-induced diabetic neuropathy rats models, Lv et al. (2018) demonstrated that J147 (a curcumin analog) elevated mechanical pain thresholds, upregulated adenosine monophosphate (AMP)-activated protein kinase (AMPK) expression, and concurrently downregulated TRPA1 levels in AITC-stimulated RSC96 Schwann cells with a better effect at 100 μM. The observed inverse correlation of AMPK and TRPA1 levels indicates that the therapeutic effects of J147 may involve AMPK-dependent suppression of TRPA1 signaling.

### 3.5 Angelicae Sinensis Radix

Angelicae Sinensis Radix (ASR), the dried root of *Angelica sinensis* (Oliv.) Diels (family: Umbelliferae), traditionally termed Danggui, is cultivated primarily in China’s Hubei and Jiangsu provinces. This botanical agent has been historically prescribed for rheumatism, gastrointestinal dysfunction, and influenza, with modern studies validating its anticancer, anti-obesity, anti-fibrotic, and anti-inflammatory properties (Jun et al., 2018; Zhi et al., 2024). Emerging evidence has further identified the ability of ASR to modulate central neurotransmission, cardiovascular homeostasis, and immune regulation (Wei et al., 2016; Chen L. et al., 2024).

The therapeutic potential extends to ASR-containing formulations. Danggui Sini Decoction (DSD), a canonical cold syndrome remedy, was evaluated by D. Zhang et al. (2019) in chronic constriction injury (CCI) rats models. DSD administration significantly attenuated cold/mechanical allodynia and thermal hyperalgesia, suppressing TRPM8, TRPV1-4, and TRPA1 protein expression in DRGs. These preliminary findings posit TRPA1 downregulation as a potential contributor to the analgesic effects of ASR, warranting mechanistic validation through targeted interrogation.

Ligustilide (Lig), a principal bioactive phthalide from ASR, displays multimodal bioactivities (Bain, 2015). Zhong et al. (2011) characterized two phthalide derivatives—Lig and its aromatized analog dehydroligustilide (DH-Lig) — through whole-cell voltage-clamp analyses in TRPA1-CHO cells. Both compounds activated TRPA1 (Lig IC_50_ = 1,456 μM and DH-Lig IC_50_ = 23 μM), although DH-Lig uniquely elicited post-washout current potentiation (off-response). Preactivation with mustard oil revealed DH-Lig’s superior inhibitory efficacy. Cysteine-substituted TRPA1 mutants demonstrated Lig’s dependence on specific residues *versus* DH-Lig’s cysteine-independent activation, indicating divergent agonistic mechanisms. Both phthalides suppressed TRPM8 while sparing TRPV1 activity. Pan et al. (2016) observed that ASR could attenuate cold-induced cutaneous vasospasm syndrome, so they studied the pharmacological interaction between Z-Ligustilide (Z-Lig) and ferulic acid (Fa), another bioactive component of the ASR. They performed isobolographic analysis and observed that the inhibition of TRPA1 and TRPM8 caused by Z-Lig and Fa was synergistic. The combination could also decrease RhoA activation, MLC20 phosphorylation, and cold-induced upregulation of TRPM8. This synergy extended to cold-induced RhoA/MLC20 signaling suppression, TRPA1 (Z-Lig IC_50_ = 6.83 ± 1.12 μM and Fa IC_50_ = 9.124 ± 1.04 μM) and TRPM8 (Z-Lig IC_50_ = 7.389 ± 1.09 μM and Fa IC_50_ = 8.318 ± 1.03 μM) inhibition, and normalization of TRPM8 expression, indicating multi-target modulation of thermoregulatory pathways.

Moreover, Ortar et al. (2013) synthesized 17 Lig/DH-Lig analogs through structure-activity relationship analyses. Notably, they found that Lig and its derivatives exhibited bidirectional regulatory effects on TRPA1 and TRPM8, indicating multiple pathways were involved in the regulations. For example, the TRPA1 EC_50_ of 5-chloro-6-methyl-3-methylenephtahilde is 4.7 ± 0.5 μM and the IC_50_ is 46.7 ± 7.2 μM. Most derivatives demonstrated enhanced TRPA1/TRPM8 modulation of the two channels with EC_50_ and/or IC_50_ values distinctly lower than those of the reference compounds. These results have provided novel scaffolds for ion channel-targeted drug discovery.

### 3.6 Faeces Bombycis

Faeces Bombycis (FB), the dried feces of *Bombyx mori* Linnaeus (commnonly known as Silk Moth, family: Bombycidae), a zoological-derived traditional remedy, has been empirically prescribed for managing cephalalgia, arthralgia, and pruritic dermatoses. Song et al. (2023) systematically evaluated FB’s anti-migraine potential through behavioral phenotyping and metabolomic profiling in rats models. Bioactivity-guided fractionation identified petroleum ether extracts as the most efficacious (reducing head-scratching behaviors). Among the seven purified metabolites obtained from petroleum ether extracts, phytol demonstrated superior channel-modulatory capacity, functioning as a multimodal ion channel antagonist that showed micromolar-range inhibition of Nav1.7, TRPV1, and TRPA1 with IC_50_ values at 0.32 ± 0.05 μM, 5.2 ± 1.1 μM and 4.6 ± 1.3 μM respectively. This 16-fold selectivity for Nav1.7 over TRP channels provides explanations for the therapeutic effect of FB on migraine and other pain-related diseases.

### 3.7 Notopterygii Radix et Rhizoma

Notopterygii Radix et Rhizoma (NRR), the dried rhizome and root of *Notopterygium incisum* Ting ex H. T. Chang or *Notopterygium franchetii* H. Boissieu (family: Umbelliferae) exerts pharmacological effects like anticancer, anti-inflammatory, immune-modulation, and bone-protection effects (Jiang et al., 2020; Ye et al., 2023; Chen J. H. et al., 2024). Using mice models of CCI- and cisplatin-induced neuropathic pain, Ruan et al. (2023) studied the effects of water extract of NRR (WN) on cold pain and found that oral administration of WN could reduce cold and mechanical allodynia in these two pain models. They also found that WN could reduce cold pain, AITC-induced pain, and the expression of TRPA1. Then, they performed calcium imaging and found that WN inhibited the AITC-induced calcium response in HEK293 cells transfected with TRPA1 and DRG neurons. They suggested that through the regulation of TRPA1, WN reduced neuropathic pain-induced cold allodynia.

### 3.8 Borneol

Borneol (BO), a bicyclic monoterpene derived from *Cinnamomum camphora* (L.) Presl (family: Lauraceae), demonstrates extensive pharmacological activities encompassing anti-inflammatory, analgesic, and antipyretic effects, with established clinical applications in cardiovascular and cerebrovascular disease management (Mei et al., 2023). Mechanistic investigations have revealed its complex interactions with TRP channels. Takaishi et al. (2013) employed calcium imaging and patch-clamp electrophysiology to quantify BO’s concentration-dependent inhibition (IC_50_ = 0.20 ± 0.06 μM) of TRPA1 activation triggered by menthol and allyl isothiocyanate (AITC) in HEK293 cells. Zhou et al. (2016) further validated this TRPA1-modulating property *in vivo*, demonstrating that intrathecal administration of (+)-BO reversed oxaliplatin-induced mechanical hyperalgesia in mice models while preserving normal motor function and body weight during a 60-µg daily treatment regimen.

Tian et al. (2023) established pruritus mice models. They identified dual antipruritic mechanisms through cutaneous BO application: TRPA1/TRPM8-dependent suppression of chloroquine-induced nonhistaminergic itch via calcium flux modulation in transfected HEK293 cells and TRPA1/TRPM8-independent inhibition of compound 48/80-mediated histaminergic itch. This functional dichotomy extends to analgesia, since Wang et al. (2017) found that the topical application of BO preserved its antinociceptive efficacy in TRPA1-knockout mice across both capsaicin-induced acute pain and CFA-induced hyperalgesia mice models. Collectively, the mechanism underlying the pharmacological effects of BO is complex, and regulation of TRPA1 accounts for only a small portion of it.

### 3.9 Puerariae Lobatae Radix

Puerariae Lobatae Radix (PLR), derived from the dried root of *Pueraria lobata* (Willd.) Ohwi (family: Fabaceae), has been extensively documented for its multifaceted pharmacological properties. Existing pharmacological evidence has confirmed its therapeutic potential in diabetes management, oncological interventions, antiviral activity, inflammatory modulation, oxidative stress mitigation, and immune system regulation (Ren and Qu, 2023). As the principal bioactive isoflavone constituent of PLR, puerarin’s neuropharmacological effects were systematically investigated by Xia et al. (2018) in a rodent neuropathic pain paradigm.

Using a neuropathic pain rat model, the research team partially ligated the sciatic nerve and administered intraperitoneal puerarin injections to evaluate its analgesic efficacy. Behavioral quantification revealed significant attenuation of mechanical allodynia and thermal hyperalgesia following puerarin intervention. Molecular analyses demonstrated dose-dependent downregulation of TRPV1 and TRPA1 mRNA overexpression after induction of neuropathy in DRG neurons. These findings mechanistically established puerarin’s capacity to ameliorate neuropathic pain through targeted suppression of TRP channel upregulation in sensory ganglia.

### 3.10 Croci Stigma

Croci Stigma (CS), the dried stigma of *Crocus sativus* L. (commonly referred to as Saffron, family: Iridaceae), demonstrates multi-target pharmacological activities, including neuroprotection, anxiolysis, depression mitigation, glycemic regulation, oxidative stress reduction, anti-inflammation, analgesia, blood pressure modulation, lipid-lowering effects, cerebral ischemia protection, and antitumor properties (Pourbagher-Shahri and Forouzanfar, 2023). The monoterpene aldehyde safranal, a principal bioactive constituent of CS, exhibits specific TRPA1 channel-mediated pharmacological actions. In a mechanistic investigation utilizing hTRPA1-HEK293 cell models, Simone Li Puma et al. (2019) employed dynamic image analysis coupled with whole-cell patch-clamp electrophysiological assessments to demonstrate concentration-dependent TRPA1 activation by safranal (EC_50_ = 9 ± 0.2 μM). This activation mechanism required three critical cysteine residues, as evidenced by the abolished responses in cysteine-deficient mutant receptors.

Safranal’s target specificity was further validated in primary rodent sensory neurons. Organ bath studies revealed concentration-dependent contractions in rat urinary bladder strips, with safranal exhibiting marginally lower potency than AITC. Neuropeptide-release assays showed dose-responsive increments in CGRP-LI flow from rat spinal cord preparations. Behavioral pharmacology experiments demonstrated safranal-induced dose-dependent acute nocifensive responses upon intraplantar administration in mice. Both TRPA1 antagonist HC-030031 and genetic ablation of TRPA1 completely abolished these pharmacological effects. Notably, safranal pretreatment induced TRPA1 desensitization, significantly attenuating subsequent AITC responses. These findings establish safranal as a novel TRPA1 partial agonist capable of producing channel desensitization, indicating a dual mechanism underlying its analgesic properties through targeted TRPA1 modulation (Li Puma et al., 2019).

In summary, a large number of TCM drugs have shown the ability to modulate TRPA1 channels, through which they can regulate pain. Among TCM drugs that are beneficial for pain-related diseases, some directly inhibit the channel, while others cause a mild activation leading to prolonged deactivation of the channel, thereby preventing activations that can produce pain. TRPA1 is strongly involved in the pathological process of neuropathic pain (Talavera et al., 2020). In this regard, the analgesic effects of TCM drugs may be related to the modulation of TRPA1, which may provide an explanation of the medical effects of these traditional herbals.

## 4 Studies on TCM drugs and their components regulating inflammation by modulating TRPA1

A growing body of literature has shown that TRPA1 is a potential target for the treatment of inflammatory disease, since it can regulate the release of the inflammatory factors such as interleukin (IL)-1 and tumor necrosis factor (TNF)-α and the functioning of immune cells such as macrophages and T cells (Yao et al., 2023). Meanwhile, some TCM drugs have been shown to possess anti-inflammatory properties, which may be partly mediated through modulation of the TRPA1 channel. Some TCM drugs have been found to show both analgesic and anti-inflammatory effects, and their mechanisms are largely interwoven and interrelated.

### 4.1 Evodiae Fructus

Evodiae Fructus (EF), also known as Euodiae Fructus, is the almost ripe fruit of *Evodia rutaecarpa* (Juss.) Benth, *E. rutaecarpa* (Juss.) Benth. var. officinalis (Dode) Huang or *E. rutaecarpa* (Juss.) Benth. var. bodinieri (Dode) Huang (family: Rutaceae). The plant is primarily distributed in various provinces and cities of China, including Jiangxi and Guangxi (Cao et al., 2018). EF, along with its related formulas such as Wu zhu-yu Decoction and Wen-jing Decoction, is renowned for its efficacy in treating digestive diseases and alleviating headaches (Yan et al., 2023). He et al. (2023a) employed a TCM-patterned irritable bowel syndrome (IBS) rats model to demonstrate the ability of the aqueous extract of EF to ameliorate weight loss/diarrhea severity, restore ATPase activity, and suppress TRPA1/TRPM8 mRNA-protein co-expression in DRG-colon axes. Rutaecarpine (Rut)—the principal quinazolinocarboline alkaloid—exerts pleiotropic effects encompassing anti-inflammatory, vasodilatory, and redox-modulatory activities (Tian et al., 2019). Pharmacological explorations of Rut have extended to synthetic derivatives: Ma et al. (2019) developed 11 rutaecarpine-furoxan hybrids, with compound 13a exhibiting dual TRPV1/TRPA1 agonism that potentiates CGRP secretion, representing a strategic approach for the development of newer antihypertensive drugs.

### 4.2 Cinnamomi Cortex

Cinnamomi Cortex (CC), also known as Cinnamon, is derived from the dried bark of *Cinnamomum cassia* Presl (family: Lauraceae), which is predominantly cultivated in China’s Guangdong and Guangxi provinces, and serves dual roles as a culinary spice and a traditional analgesic/thermogenic agent (Liu et al., 2021). Pharmacological profiling has revealed its multifunctional therapeutic potential: glycemic control through insulin modulation (Chen G. et al., 2013), antiviral defense (Fujikane et al., 2022), antithrombotic activity (Ozhan et al., 2023), antitumor effects (Bae et al., 2015), and cardioprotection against ischemia-reperfusion injury (Liu et al., 2023).

Mechanistic studies by Xiang et al. (2021) employed high-performance liquid chromatography (HPLC)-quadrupole time of flight (Q-TOF)/mass spectrometry (MS) metabolomics with pathway enrichment analysis to delineate CC’s thermoregulatory actions. Three principal constituents enhanced lipolytic metabolism and brown adipocyte activation via upregulation of UCP1, PGC1-α, and pHSL expression, concomitantly stimulating TRPA1 in DRG neurons.

Cinnamaldehyde (CNA) is an essential oil extracted from CC (Muhoza et al., 2023) and is responsible for the efficacy and bioactivity of CC (Zhu et al., 2017; Zhang et al., 2023). Kakae et al. (2023) used a vascular cognitive impairment (VCI) mice model and observed that CNA could improve the cognitive function and myelin density of the model, and that these inhibitory effects diminished significantly in *TRPA1* gene-knockout mice. This suggests that CNA can ameliorate VCI through regulation of TRPA1. Chen et al. (2021) observed that CC and CNA could preserve the expression of TRPA1 in a overreactive bladder (OAB) mice model. They used cyclophosphamide (CYP) to induce typical OAB pathophysiological changes in the model mice, and TRPA1 expression was found to be downregulated in model mice. However, treatment with CC and CNA significantly ameliorated the CYP-induced dysfunction in micturition patterns and pathophysiological changes and suppressed local inflammation, while preserving the expression of TRPA1. However, administration of HC-030031 (a TRPA1 agonist) did not cause significant improvement in CYP-induced abnormal micturition patterns. Thus, the ameliorative effects of CNA on VCI are mediated through the regulation of TRPA1, while in the case of OAB, the preservation of TRPA1 expression by CC and CNA appears to be beneficial, although direct activation of TRPA1 was not found to be involved in the amelioration.

### 4.3 Alpiniae Officinarum Rhizoma

Alpiniae Officinarum Rhizoma (AOR), also known as Lesser Galangal, is derived from the dried rhizome of *Alpinia officinarum* Hance (family: Zingiberaceae), which is indigenously cultivated in subtropical regions of southern China and Vietnam (Abubakar et al., 2018), and possesses broad-spectrum pharmacological properties including antioxidant, antidiabetic, and antiemetic activities (Ahmad et al., 2017). He et al. (2023b) used an IBS mice model to demonstrate AOR aqueous extract’s therapeutic capacity: ameliorating weight loss/diarrhea severity, restoring ATPase functionality, and suppressing TRPA1/TRPM8 mRNA-protein co-expression along the DRG-colon axis. These multimodal effects provide mechanistic linkage to AOR’s traditional thermogenic and analgesic properties through dual regulation of TRP channel homeostasis.

### 4.4 Zingiberis Rhizoma

Zingiberis Rhizoma (ZR), which is derived from the dried rhizome of *Zingiber officinale* Roscoe (commonly referred to as Ginger, family: Zingiberaceae), exhibits therapeutic potential across multiple disease domains. Clinically applied for gastrointestinal disorders such as nausea, vomiting, abdominal discomfort, and diarrhea, ZR has also demonstrated efficacy in arthritis, rheumatism, musculoskeletal pain, cardiovascular conditions, and metabolic dysregulation (de Lima et al., 2018). The traditional Chinese medicine formula Da-Jian-Zhong Decoction (DJZD), which incorporates ZR as a key component, shows notable activity against abdominal pain (Li C. et al., 2023; Zhang et al., 2024).

Endo et al. (2017) elucidated the pivotal role of ZR in mediating DJZD’s anti-inflammatory effects using a mice postoperative ileus model. Their investigation revealed ZR’s dual capacity to enhance intestinal motility and reduce CD68-positive macrophage infiltration within ileal muscular layers. Pharmacological interventions with HC-030031 attenuated both ZR/DJZD-induced anti-inflammatory responses and 5-hydroxytryptamine (5-HT) secretion from RIN-14B cells exposed to gingerols/shogaols, indicating that TRPA1 channel activation in enterochromaffin (EC) cells was the mechanistic basis for macrophage suppression. Subsequent research employing analogous methodologies further demonstrated TRPA1-mediated prevention of neutrophil infiltration by these agents (Endo et al., 2018).

The existing evidence has identified zingerone, shogaols and gingerols as the principal bioactive constituents of ZR (Kim et al., 2016; Zhang et al., 2018). Huang et al. (2019) documented 6-shogaol’s capacity to induce calcium influx and generate action potentials in nodose C-fibers through TRPA1 activation, with subsequent application desensitization specific to 6-shogaol but not mechanical stimuli. Patil et al. (2022) established that vincristine-induced vagal nociceptor activation in upper gastrointestinal tissues requires TRPA1/5-HT3 receptor co-activation, a process effectively inhibited by 6-shogaol. Complementary cellular studies by Yang et al. (2016) demonstrated gingerol-induced calcium mobilization and subsequent serotonin/cholecystokinin secretion in RIN14B/STC-1 cells, which was completely abrogated by TRPA1 antagonists (ruthenium red), calcium chelation (EGTA), and Trpa1 gene silencing. These findings collectively implicate the potential of ZR-derived compounds in modulating gastrointestinal function through TRPA1-dependent endocrine signaling pathways.

### 4.5 Schisandrae Chinensis Fructus

Schisandrae Chinensis Fructus (SCF), also known as Schisandra Berry, which is derived from the dried ripe fruit of *Schisandra chinensis* (Turcz.) Baill. (family: Magnoliaceae), exhibits diverse pharmacological properties encompassing antioxidant, anti-inflammatory, anti-osteoporotic, antidepressant, and antitussive effects (Li et al., 2018; Yuan et al., 2018; Fu et al., 2023). In a seminal study by Zhong et al. (2015), a cigarette smoke-induced cough hypersensitivity model was established in guinea pigs. Both ethanol extracts (EEs) and ethanol-water extracts (EWEs) of SCF demonstrated significant efficacy in reducing 0.4M citric acid-evoked cough frequency. Notably, pretreatment with these extracts markedly attenuated cigarette smoke-induced pulmonary neutrophilic infiltration and total inflammatory cell accumulation, concurrently suppressing elevated pulmonary malondialdehyde, TNF-, and IL-8 levels. Parallel enhancements in pulmonary superoxide dismutase (SOD) and glutathione (GSH) activities were observed, highlighting the extracts’ antioxidative capacity.

Histopathological analysis revealed that 1 g/kg EE pretreatment substantially mitigated airway epithelial hyperplasia, smooth muscle thickening, and inflammatory cell infiltration. Mechanistically, this treatment downregulated TRPV1 and TRPA1 expression in pulmonary tissues. Complementary *in vitro* investigations using A549 cells demonstrated that four specific *Schisandra* lignans from the EE dose-dependently inhibited cigarette smoke extract-induced TRPV1, TRPA1, and endothelial nitric oxide synthase (NOS3) expression, while concurrently suppressing nitric oxide (NO) release. These findings collectively elucidate the multimodal therapeutic mechanisms of SCF in respiratory pathophysiology.

### 4.6 Zanthoxyli Pericarpium

Zanthoxyli Pericarpium (ZP), also known as Sichuan Pepper, is derived from the dried pericarp of mature fruits from *Zanthoxylum schinifolium* Sieb. Et Zucc. or *Zanthoxylum bungeanum* Maxim. (family: Rutaceae), and has been pharmacologically characterized for its anti-inflammatory, analgesic, antioxidant, antitumor, antimicrobial, and insecticidal properties (Qi et al., 2024). In a study by Zhou et al. (2022), chronic eczema was induced in a rat model via 2-dinitrochlorobenzene (DNCB) sensitization. Comparative gas chromatography (GC)-MS analysis of volatile oil constituents between ZP and *Z. schinifolium* (ZS) revealed distinct compositional profiles.

Both medium-dose ZP and ZS interventions significantly improved the eczema area and severity index (EASI) values, which are a validated metric of antipruritic efficacy, while reducing scratching frequency. These treatments concurrently suppressed serum concentrations of IL-4, IgE, gastrin-releasing peptide (GRP), and histamine, in addition to downregulating protein expression of histamine H1 receptor (H1R), protease-activated receptor 2 (PAR-2), TRPV1, and gastrin-releasing peptide receptor (GRPR). Notably, while TRPA1 protein expression remained unchanged between model and blank controls, all ZP dosage groups and the ZS group exhibited significant TRPA1 upregulation. Paradoxically, TRPA1 mRNA levels were elevated in model animals but markedly reduced following ZP/ZS administration across all tested doses. This discordance between transcriptional and translational regulation of TRPA1 highlights the need for mechanistic investigations into the modulatory effects of ZP on this molecular target.

### 4.7 Paeoniae Radix

Paeoniae Radix Alba (white peony root) and Paeoniae Radix Rubra (red peony root), botanically derived from the dried roots of *Paeonia lactiflora* Pall. or *Paeonia veitchii* Lynch (commonly known as Chinese Peony, family: Ranunculaceae), are pharmacologically analogous herbs collectively termed Paeoniae Radix (PR) in traditional Chinese medicine. PR and its bioactive constituents exhibit multifaceted therapeutic properties, including anti-inflammatory, immunoregulatory, antitumor, antimicrobial, antioxidant, hepatoprotective, and neuroprotective activities (Yan et al., 2018). Paeoniflorin (PAE), the principal active glycoside isolated from PR, demonstrates significant neuromodulatory effects (Zhang and Wei, 2020). In a neuropathic pain model, Zhang et al. (2022) demonstrated that oral PAE administration attenuated mechanical allodynia through multiple pathyways: downregulating TRPM8, TRPV1, and TRPA1 expression in DRGs, and suppressing p38 mitogen-activated protein kinase (MAPK) phosphorylation and proinflammatory cytokine release. *In vitro* experiments further revealed PAE’s capacity to inhibit H_2_O_2_-induced TRPA1/TRPV1 upregulation and p38MAPK activation in Schwann cells, indicating a reactive oxygen species (ROS)-mediated pathway modulation (Zhang D. et al., 2021). Zhan et al. (2021) investigated PAE’s gastrointestinal effects using a loperamide-induced constipation model. PAE treatment normalized intestinal motility, reduced histopathological colonic damage, and enhanced enterochromaffin cell function through Tph1-dependent 5-HT biosynthesis. Mechanistically, PAE upregulated TGR5 receptor expression and activated PLC-γ1/PIP2 signaling cascades in RIN-14B cells, with TRPA1 channel involvement confirmed by TGR5 inhibition experiments These findings collectively elucidate PAE’s dual regulatory role in neuronal sensitization and enteric neurotransmitter regulation.

### 4.8 Rhei Radix et Rhizoma

Rhei Radix et Rhizoma (RRR), commonly referred to as Rhubarb, is derived from the dried roots of *Rheum palmatum* L., *Rheum tanguticum* Maxim. Ex Balf., or *Rheum officinale* Baill. (family: Polygonaceae). Contemporary pharmacological studies have demonstrated that RRR exhibits multifaceted bioactivities, including antitumor effects, modulation of gastrointestinal microbiota, intestinal mucosal barrier protection, anti-inflammatory properties, and antifibrotic potential (Xiang et al., 2020). Notably, Li et al. (2021) systematically investigated the hepatotoxic profile of RRR through a comprehensive approach. Using ultra-high-performance liquid chromatography (UPLC)-Q-TOF/MS technology, the researchers first characterized the chemical constituents in RRR extracts. Subsequently, three dose cohorts and a control group of rats received oral administration of these extracts, with plasma samples collected for metabolomic profiling. Through principal component analysis (PCA) and partial least squares discriminant analysis (PLS-DA), nine distinct metabolic biomarkers associated with RRR-induced hepatotoxicity were identified.

The investigation further employed computational target prediction to map hepatotoxic metabolites against known RRR components. Gene ontology (GO) enrichment and Kyoto Encyclopedia of Genes and Genomes (KEGG) pathway analyses revealed potential molecular mechanisms, implicating key targets such as DRD1, DRD2, PDE4B, TRPV1, TRPM8, TRPA1, PTGER2, ACHE, and CHRM3. These findings suggest that RRR-induced hepatotoxicity may involve dysregulation of cyclic AMP (cAMP) signaling, cholinergic synaptic transmission, and TRP channel-related inflammatory mediators. This mechanistic elucidation provides critical insights into the dual pharmacological and toxicological characteristics of RRR.

### 4.9 Glycyrrhizae Radix et Rhizoma

Glycyrrhizae Radix et Rhizoma (GRR), the dried root and rhizome derived from *Glycyrrhiza uralensis* Fisch., *Glycyrrhiza inflata* Bat., or *Glycyrrhiza glabra* L. (commonly known as Licorice or Liquorice, family: Fabaceae), has been pharmacologically demonstrated to possess multifunctional therapeutic properties. The existing research has confirmed its broad-spectrum bioactivities encompassing antitumor, antimicrobial, antiviral, anti-inflammatory, antidiabetic, immunomodulatory, hepatoprotective, and neuroprotective effects, along with adrenal cortical hormone-mimetic properties (Yang et al., 2017). Liquiritin (LIQ) is the main flavonoid compound and active ingredient of GRR.

Through whole-cell patch-clamp electrophysiology, Liu et al. (2020) identified LIQ’s bidirectional inhibitory effects on agonist-activated TRPV1 (IC_50_ = 9.8 ± 1.5 μM) and TRPA1 (IC_50_ = 16.2 ± 1.1 μM) currents. In lipopolysaccharide (LPS)-induced acute lung injury mice models, oral LIQ administration demonstrated multimodal protective efficacy: attenuating pulmonary histopathological damage, suppressing inflammatory responses, and inhibiting both nuclear factor (NF)-κB signaling activation and LPS-triggered upregulation of TRPV1/TRPA1 expression at translational (lung tissue proteins) and transcriptional (bronchoalveolar lavage fluid mRNA) levels. Parallel *in vitro* experiments using THP-1 monocytes revealed LIQ’s capacity to counteract cytokine-mediated NF-κB activation and concomitant TRPV1/TRPA1 overexpression, mirroring the effects of pharmacological antagonists. These findings establish a mechanistic link between LIQ’s anti-inflammatory/antitussive actions and its dual-channel inhibition of NF-κB-dependent TRPV1/TRPA1 upregulation in non-neuronal cells, effectively disrupting the proinflammatory positive feedback loop in airway inflammation.

### 4.10 Lycii Fructus

Lycii Fructus (LF), also known as Goji Berry, derived from the dried ripe fruits of *Lycium barbarum* L. (family: Solanaceae), is a TCM herb primarily used to tonify the liver and kidney while enhancing ocular function. Existing pharmacological investigations have demonstrated its multifunctional properties, including antioxidant, antimicrobial, immunomodulatory, and anti-inflammatory activities (Teixeira et al., 2023). Polysaccharides, identified as the principal bioactive constituents of LF, are considered critical mediators of its therapeutic efficacy (Xiao et al., 2022). In a mechanistic study by Chen et al. (2022), dextran sulfate sodium (DSS)-induced ulcerative colitis (UC) rat models were orally administered *L. barbarum* polysaccharides (LBP). This intervention significantly attenuated serum malondialdehyde levels and IL-6 concentrations, while concurrently elevating catalase activity. At the colonic level, LBP administration substantially reduced TNF-α expression and downregulated TRPV1 and TRPA1 channel protein expression. Notably, co-administration of LBP with capsaicin demonstrated synergistic effects, exhibiting enhanced suppression of serum IL-6 and colonic TNF-α levels, along with more pronounced inhibition of TRPA1 protein expression in comparison with monotherapy. These findings collectively indicate LBP-mediated modulation of oxidative stress and inflammatory pathways in UC pathophysiology.

### 4.11 Platycodi Radix

Platycodi Radix (PlaR), the desiccated root system of *Platycodon grandiflorum* (Jacq.) A. DC. (commonly referred to as Balloon Flower, family: Campanulaceae), demonstrates multi-pharmacological efficacy, including expectorant, antitussive, anti-inflammatory, antineoplastic, anti-obesity, hypoglycemic, immunoregulatory, cardiovascular protective, and hepatoprotective properties (Zhang L. L. et al., 2020). In a lipopolysaccharide (LPS)-induced pneumonia mice model established by Yang et al. (2023), PlaR treatment exhibited the following dose-dependent therapeutic effects: (1) alleviation of LPS-induced pulmonary histopathological alterations; (2) suppression of the production of the proinflammatory cytokines TNF-α and IL-1β; and (3) downregulation of TRPA1 mRNA and protein overexpression. Notably, high-dose PlaR administration induced modest TRPA1 upregulation in healthy controls, although the upregulation was significantly less pronounced than LPS-induced activation. They also identified five primary platycodins in PlaR water extract, and Platycodin D3 showed the highest affinity for TRPA1 through molecular docking and experimental validation.

### 4.12 Chuanxiong Rhizoma

Chuanxiong Rhizoma (CR) is the desiccated rhizome of *Ligusticum chuanxiong* Hort. (family: Apiaceae). Contemporary pharmacological investigations have demonstrated that CR possesses multifunctional therapeutic properties, including antioxidant activity, anti-inflammatory effects, ischemic injury protection ability, and immunomodulatory capacity, with clinical applications in dysmenorrhea and diabetes management (Rai et al., 2019; Wang et al., 2020). Ligustrazine, identified as the principal bioactive compound mediating the therapeutic efficacy of CR (Zou et al., 2018), was systematically investigated by Yu et al. (2022) using a mice model of chronic venous disease (CVeD)-induced nociception. Their experimental design revealed that ligustrazine administration significantly attenuated mechanical allodynia and thermal/cold hyperalgesia induced by venous ligation.

Through electrophysiological patch-clamp recordings and calcium imaging analyses, the researchers elucidated ligustrazine’s capacity to desensitize TRPA1 channels in DRG neurons. The compound demonstrated additional therapeutic mechanisms by suppressing macrophage infiltration and downregulating mRNA expression levels of IL-1β and NLRP3 inflammasome components in DRG tissues. Notably, while these therapeutic effects were observed *in vivo*, *in vitro* experiments using TRPA1-transfected HEK293 cells showed no direct channel blockade. This dichotomy indicates that ligustrazine’s antinociceptive action in CVeD primarily involves indirect modulation of TRPA1 activity through inhibition of inflammatory pathways rather than direct receptor antagonism.

Notably, ligustilide, another pharmacologically significant constituent of CR(Chen et al., 2018), exhibits contrasting TRPA1 activation properties as detailed in Section 3.5, highlighting the complex pharmacodynamics of CR’s multi-component system.

### 4.13 Sophorae Flavescentis Radix

Sophorae Flavescentis Radix (SFR), the dried root of *Sophora flavescens* Ait. (family: Fabaceae Lindl.), has been demonstrated through contemporary pharmacological studies to exhibit multi-target therapeutic properties, including physiological regulation, anti-aging, antitumor, anti-inflammatory, and immunomodulatory effects (Chen et al., 2020; Sun P. et al., 2022). Using computational approaches combining data mining with bioinformatics, Zhong et al. (2017) suggested that TRPA1 was the convergent molecular target of SFR, Fructus Cnidii (FC), and pruritus pathogenesis. GO and KEGG analyses further identified calcium ion homeostasis and the calcium signaling pathway as critical biological processes within this target network, indicating the central role of TRPA1 in mediating the antipruritic effects of SFR and FC.

Oxymatrine (OMT), a quinolizidine alkaloid isolated from SFR, demonstrates broad pharmacological activity (Li et al., 2020). Zhang et al. (2021a) employed DRG-cell membrane immobilized chromatography to identify five bioactive SFR compounds. Using four distinct pruritus models (chloroquine [CQ], Ser-Leu-Ile-Gly-Arg-Leu [SLIGRL], histamine [HIS], and AITC), they observed that OMT significantly attenuated scratching behavior in CQ- and SLIGRL-induced mice models (histamine-independent pathways), and its dose-dependent efficacy was correlated with TRPA1 mRNA downregulation. Notably, OMT also suppressed AITC-induced nociceptive responses. In a complementary study by Zhu et al. (2020) using a squaric acid dibutyl ester (SADBE)-induced allergic contact dermatitis (ACD) murine model, all three OMT formulations (gel, microgel powder, microgel cream) effectively reduced facial wiping and scratching episodes while mitigating cutaneous inflammation. The optimized oxymatrine microgel cream (OMC) formulation exhibited superior therapeutic outcomes, including restoration of epidermal keratinization patterns, reduction of peripheral leukocytosis, and systemic downregulation of proinflammatory mediators (IL-1β, TNF-α, CXCR3, CXCL10, IL-6, IL-10, IL-17A, IL-31), along with suppression of TRPA1/TRPV1 expression in trigeminal ganglia and cytokine modulation in thymic interferon (IFN)-γ and splenic IL-17A tissues. Comparative analysis established OMC as the most effective dosage form through multimodal mechanisms involving chemokine regulation, immune mediator modulation, and TRP channel inhibition.

### 4.14 Tetrapanacis Medulla

Tetrapanacis Medulla (TM), the dried stem pith of *Tetrapanax papyrifer* (Hook.) K. Koch (commonly known as Rice-paper Plant, family: Araliaceae), serves as a traditional botanical source of hederagenin. This pentacyclic triterpenoid demonstrates diverse pharmacological properties, including antitumor, anti-inflammatory, antidepressant, anti-neurodegenerative, antihyperlipidemic, antidiabetic, anti-leishmaniasis, and antiviral activities (Huang et al., 2023). Zhang et al. (2020) used KEGG pathway analysis and identified “inflammatory mediator regulation of TRP channels” as the most significantly enriched pathway. Subsequent experiments utilizing a CCI rat model revealed that both oral TM administration and hederagenin treatment effectively alleviated the manifestations of neuropathic pain, specifically mechanical/thermal hyperalgesia, cold allodynia, and tactile hypersensitivity. Biochemical analyses demonstrated TM’s capacity to significantly reduce serum concentrations of proinflammatory cytokines (IL-1β, IL-6, TNF-α). At the molecular level, TM and hederagenin coordinately downregulated the expressions of multiple transient receptor potential channels (TRPM8, TRPA1, TRPV1, TRPV4) in DRGs and inhibited phosphorylation of p38 MAPK.

In general, TCM drugs can modulate TRPA1 as part of a complex, multimodal mechanism to suppress the release of inflammatory factors and macrophage infiltration, with TRPA1 being one of several contributing targets. Some of them downregulate TRPA1 protein and mRNA expression, while some others preserve the expression reduced by some pathological process. We have also noticed that herbal drugs such as ZP modulate the expression of mRNA and protein oppositely, and herbal drugs such as PR and CR activate and inhibit the channel under different conditions. These diverse modulations of TRPA1 indicate the need for more studies to characterize the anti-inflammatory effects of TCM drugs.

## 5 Studies on TCM drugs and their components with agonistic or inhibitory effects on TRPA1

Some TCM drugs and their ingredients have been found to be TRPA1 agonists/antagonists, and their activities are mostly accomplished by monitoring intracellular Ca^2+^ levels. However, the metabolic pathways and pathological processes underlying these TCM drugs and their ingredients remain to be elucidated.

### 5.1 Piperis Fructus

Piperis Fructus (PF), the fruits of *Piper nigrum* L. (commonly referred to as Pepper, family: Piperaceae), which has been globally utilized as a culinary spice and phytotherapeutic agent, demonstrates a multifaceted pharmacological profile that includes antimicrobial, neuroprotective, and immunoregulatory activities (Zheng et al., 2016; Takooree et al., 2019). Yukiko Okumura et al. (Okumura et al., 2014) systematically characterized 19 PF-derived alkaloids using heterologously expressed human TRPV1/TRPA1-HEK293 models. Notably, 18 of the 19 compounds evoked concentration-dependent intracellular Ca^2+^ mobilization through both channel subtypes, with complete response attenuation by specific antagonists (AMG9810 for TRPV1, HC-030031 for TRPA1). Mechanistically, piperine/isopiperine emerged as dual TRPV1/TRPA1 agonists, providing molecular rationale for the traditional medicinal applications of PF.

### 5.2 Allii Sativi Bulbus

Allii Sativi Bulbus (ASB), also known as Garlic, is the bulb of *Allium sativum* L. (family: Liliaceae). It is a globally consumed culinary-medicinal bulb that has been empirically utilized in TCM for its thermogenic, detoxicant, and anti-inflammatory properties (Ahmed and Wang, 2021). These properties are attributed to its antioxidant, immunomodulatory, and metabolic regulatory activities (Kumari et al., 2022). Koizumi et al. (2009) quantified intracellular Ca^2+^ flux in TRPA1/TRPV1-CHO models using organosulfur compounds (diallyl sulfide/disulfide/trisulfide), demonstrating dual-channel agonist activity with preferential TRPA1 activation with EC_50_ at 254, 7.55 and 0.49 μM respectively. Macpherson et al. (2005) corroborated these findings through fluorescent imaging plate reader (FLIPR)-based calcium imaging, and their findings showed ASB extract-induced dose-dependent activation of both channels. Allicin has emerged as the predominant activator (EC_50_ for mTRPA1, hTRPA1 and rTRPV1 were 1.32 μM, 1.91 μM, and 51.22 μM), with methodological assessments using comparative FLIPR-nuclear magnetic resonance (NMR) quantification confirming allicin’s concentration-dependent pungency. These convergent results establish allicin as the primary mediator of ASB’s TRP channel-mediated bioactivity.

### 5.3 Alpiniae Katsumadai Semen

Alpiniae Katsumadai Semen (AKS), also known as Katsumada’s Galangal, which is derived from the dried near-mature seeds of *Alpinia katsumadai* Hayata (family: Zingiberaceae), exhibits multimodal bioactivities, including antioxidant, antineoplastic, and nociception-modulating effects (Li et al., 2012; Park et al., 2014). Cardamonin, the principal chalcone constituent, mediates the bioactivities of AKS through pro-apoptotic, cell cycle-modulatory, and autophagy-inducing mechanisms (Nawaz et al., 2020).

Mechanistic insights from the study by Wang et al. (2016) identified cardamonin’s selective TRPA1 antagonism via calcium influx assays complemented by molecular docking. This chalcone demonstrated concentration-dependent TRPA1 blockade (IC_50_ = 454 nM) with structural compatibility at the A-967079-binding pocket, while remaining inert toward TRPV1/V4. Cytotoxicity profiling (CellTiter-Glo/xCELLigence RTCA systems) confirmed preserved HEK293 viability and unaltered cardiomyocyte contractility, validating its therapeutic safety window.

### 5.4 Asari Radix et Rhizoma

Asari Radix et Rhizoma (ARR), also known as Wild Ginger, is the dried root and rhizome of *Asarum heterotropoides* Fr. Schmidt var. Mandshuricum (Maxim.) Kitag., Miq. var. Seoulense Nakai or *Asarum sieboldii* Miq (family: Aristolochiaceae). It is a botanical source of analgesic, anticonvulsant, and anti-inflammatory agents, and has been integrated into traditional formulations such as Mahuang Fuzi Xixin Decoction (MFXD). Network pharmacology analysis by Jiang et al. (2021) identified TRPA1 as the convergent molecular target linking ARR’s bioactive components with migraine pathophysiology. ARR is also present in numerous TCM formulas, such as MFXD. Matsumoto et al. (2020) performed qualitative non-targeted analysis utilizing high-resolution MS to identify the bioactive ingredients of ARR, and quantitative targeted analysis to identify the plasma concentrations following oral administration of maobushisaishinto (MFXD in Kampo medicine) in rats. Then, they performed *in vitro* pharmacological studies of bioactive ARR ingredients in addition to other ingredients of MFXD to reveal that methyl kakuol (EC_50_ = 0.27 μM), Amide A (EC_50_ = 0.47 μM), Asarinin (EC_50_ = 3.1 μM), Sesamin (EC_50_ = 2.3 μM) and other compounds derived from ARR were detected in rat plasma and showed agonistic activity on the TRPA1 channel. Their research indicates that methyl kakuol and other compounds may be novel TRPA1 agonists and may contribute in part to the bioactive effects of ARR and MFXD.

### 5.5 Perillae Fructus/Perillae Folium

Perillae Fructus/Folium (PFB), which consists of the dried ripe fruits and foliar young branches of *Perilla frutescens* (L.) Britt. (commonly referred to as Perilla, family: Lamiaceae), exhibits broad-spectrum bioactivities, including antioxidant, anticancer, and antimicrobial effects (Hou et al., 2022). Angela Bassoli et al. (2009) characterized two principal sesquiterpenoids, perillaldehyde (EC_50_ = 41.0 ± 7.5 μM) and perillaketone (EC_50_ = 19.7 ± 1.7 μM), as potent TRPA1 agonists in heterologous-expression systems. Building on these lead compounds, subsequent structure-activity relationship studies (Bassoli et al., 2013; Cattaneo et al., 2017) yielded novel synthetic analogs with enhanced TRPA1 activation profiles. These findings collectively establish PFB as a valuable phytochemical repository for developing TRP channel-targeted therapeutics.

### 5.6 *Waldheimia glabra* (Decne.) Regel

*Waldheimia glabra* (Decne.) Regel (WR), a perennial herb belonging to the Asteraceae family and endemic to the Himalayan Mountains, has been employed in ethnomedicine for millennia to manage dermatological conditions, cephalalgia, arthralgia, and pyrexia. Giorgi et al. (2017) performed headspace solid-phase microextraction (HS-SPME) GC/MS, GC/MS, and NMR analyses to identify the two main components of WR: (+)-Ludartin (EC_50_ = 36.9 ± 5.7 μM) and B-ring-homo-tonghaosu (EC_50_ = 40.4 ± 3.8 μM) (compound 1 and 2). They then tested the efficacy of WR extracts and two compounds in the TRPA1-or TRPM8-transfected HEK293 cells. Although all tested extracts and compounds exhibited minimal inhibitory effects on rat TRPM8 channels, significant agonist activity was observed at TRPA1 receptors. Notably, both compounds demonstrated relatively low potency as TRPA1 agonists. Of particular structural interest, compound 2 represents a previously uncharacterized spirocyclic scaffold in TRPA1 agonist pharmacology, indicating its potential utility as a lead compound for developing novel TRPA1-targeting therapeutics.

### 5.7 *Kalopanax pictus* Nakai

*Kalopanax pictus* Nakai (KPN), a deciduous tree belonging to the Araliaceae family, is predominantly distributed in East Asia. The dried leaves and bark of this species have been historically employed for the management of diverse pathological conditions in traditional medicine across China and neighboring Asian countries. Contemporary pharmacological investigations have revealed that KPN exhibits multifaceted therapeutic potential, including anti-inflammatory, antinociceptive, antidiabetic, and anticancer properties (Park et al., 2022).

In a pivotal study by Son et al. (2012b), calcium imaging analyses were performed on cultured cells expressing human TRPA1 (hTRPA1) and TRPV1 (hTRPV1) channels. Their findings demonstrated that 80% EEs derived from KPN leaves induced time- and concentration-dependent intracellular Ca^2+^ influx through both hTRPA1 and hTRPV1 activation. Notably, this TRPA1-mediated response was significantly attenuated by co-treatment with ruthenium red (a broad-spectrum TRP channel antagonist) and HC-030031 (a selective TRPA1 inhibitor). Subsequent pharmacological evaluation of six bioactive compounds isolated from KPN revealed that methyl syringate exhibited selective agonistic activity toward hTRPA1 (EC_50_ = 507.4 µM), while showing negligible effects on hTRPV1. These results established methyl syringate as a novel natural product with specific TRPA1-activating properties (Son et al., 2012a).

## 6 Conclusions and perspectives

Our review provides a summary of the TCM drugs that act on TRPA1 to enhance the understanding of the pharmacological effects of TCM drugs and to provide evidence for further research on the molecular basis of novel drug development and modern application of TCM. In addition to the TCM drugs and ingredients mentioned above, some other compounds extracted from TCM drugs and formulas have also been shown to act on TRPA1, and the information regarding these herbs and their ingredients is comprehensively summarized in Table 1. Some other TCM formulas have also been proven to regulate TRPA1 through modern pharmacological research, and these formulas are listed in Table 2.

**TABLE 1:** List of TCMs and related compounds that regulate TRPA1.

Name of the drug	Species tested	Pharmacologic effect	Regulatory effect on TRPA1	Compounds and potencies	References
Aconiti Radix (AR)	Mouse	Alleviates CIBP-induced cold allodynia and mechanical allodynia	Inhibits activity and reduces protein expression	Aconitine (not determined)	Wang et al. (2015), Zhou et al. (2020), Wu et al. (2021b), Gao et al. (2022), He et al. (2023a), Jin et al. (2023)
Evodiae Fructus (EF)	Rat	Reduces inflammatory cell infiltration	Suppresses protein and mRNA expression	Rutaecarpine (not determined)	Ma et al. (2019), Tian et al. (2019), He et al. (2023b), Yan et al. (2023)
Cinnamomi Cortex (CC)	Mouse	Preserves the structure of the bladder tissue from inflammation	Preserves expression	Cinnamaldehyde (not determined)	Chen et al. (2013a), Bae et al. (2015), Zhu et al. (2017), Chen et al. (2021), Liu et al. (2021), Fujikane et al. (2022), Kakae et al. (2023), Liu et al. (2023), Muhoza et al. (2023), Ozhan et al. (2023), Zhang et al. (2023)
Alpiniae Officinarum Rhizoma (AOR)	Moue	Reduces the infiltration of inflammatory cells	Suppress protein and mRNA expression	not determined	Ahmad et al. (2017), Abubakar et al. (2018), He et al. (2023b)
Piperis Fructus (PF)	Human	Agonist	Upregulates protein expression and activates the channel	Piperine (EC_50_ = 29.7 μM) (Emax = 77%) Isopiperine (EC_50_ = 32.6 μM) (Emax = 84.8%) Isochavicine (EC_50_ = 71.1 μM) Piperanine (EC_50_ = 148.3 μM) Piperolein A (EC_50_ = 7.8 μM) (Emax = 89.9%) Piperolein B (EC_50_ = 11.1 μM) (Emax = 70.4%)	Okumura et al. (2014), Zheng et al. (2016), Takooree et al. (2019)
Zingiberis Rhizoma (ZR)	Mouse	Reduces CD68-positive macrophage infiltration	Activates the channel	6- shogaol (not determined) Gingerol (not determined) Zingerone (not determined)	Kim et al. (2016), Yang et al. (2016), Endo et al. (2017), de Lima et al. (2018), Endo et al. (2018), Zhang et al. (2018), Huang et al. (2019), Patil et al. (2022), Li et al. (2023a), Zhang et al. (2024)
Camphor	Mouse	Reduces nociceptive behavior	Inhibits activity and reduces protein expression	Camphor (IC_50_ = 1.26 ± 0.32 mM)	Xu et al. (2005), Chen et al. (2013c), Takaishi et al. (2013), Li et al. (2023b)
Atractylodis Rhizoma (AtR)	Mouse Rat	Evokes moderate and prolonged nociceptive behavior; accelerates the recovery of model rats’ body surface temperature, rectal temperature, and blood flow after the cold water immersion	Activates the channel	Atractylodin, (EC_50_ = 0.913 μM) atractylodin carboxylic acid (EC_50_ = 4.4 μM)	Koonrungsesomboon et al. (2014), Wei et al. (2017), Jun et al. (2018), Kanda et al. (2021), Shimizu et al. (2022)
Allii Sativi Bulbus (ASB)	Mouse Human	Agonist	Activates the channel	Diallyl sulfide (EC_50_ = 254 μM) (Emax = 90.8%) Diallyl disulfide (EC_50_ = 7.55 μM) (Emax = 76.6%) Diallyl trisulfide (EC_50_ = 0.49 μM) (Emax = 83.6%) Allicin (mTRPA1 EC_50_ = 1.32 μM) (hTRPA1 EC_50_ = 1.91 μM)	Macpherson et al. (2005), Koizumi et al. (2009), Ahmed and Wang (2021), Kumari et al. (2022)
Curcumae Longae Rhizoma (CLR)	Rat	Reduces the mechanical withdrawal threshold in a rat model	Downregulates expression and inhibits activation	Curcumin (IC50 = 4.37 μM) Demethoxycurcumin (IC50 = 7.35 μM)	Kocaadam and Şanlier (2015), Lv et al. (2018), Zhang and Kitts (2021), Peng et al. (2022)
Angelicae Sinensis Radix (ASR)	Rat	Reduces cold allodynia, mechanical allodynia, and thermal hyperalgesia	Activates and inhibits under different conditions	Ligustilide (IC_50_ = 1,456 μM) (EC_50_ = 44 μM) Dehydroligustilide (IC_50_ = 23 μM) Z-Ligustilide (IC_50_ = 6.83 ± 1.12 μM) Ferulic Acid (IC_50_ = 9.124 ± 1.04 μM)	Zhong et al. (2011), Ortar et al., 2013; Bain (2015), Pan et al. (2016), Wei et al. (2016), Zhang et al. (2019), Shimizu et al. (2022), Chen et al. (2024b), Zhi et al. (2024)
Faeces Bombycis (FB)	Rat	Reduces migraine behaviors (head scratching)	Inhibits activity and reduces protein expression	Phytol (IC_50_ = 0.32 ± 0.05 μM)	Song et al. (2023)
Alpiniae Katsumadai Semen (AKS)	not determined	Antagonist	Inhibits activity and reduces protein expression	Cardamonin (IC_50_ = 454 nM)	Li et al. (2012), Park et al. (2014), Wang et al. (2016), Nawaz et al. (2020)
Chuanxiong Rhizoma (CR)	Mouse	Restricts macrophage infiltration and decreases the mRNA levels of IL-1β	Activates and inhibits under different conditions	Ligustrazine (not determined) Ligustilide (IC_50_ = 1,456 μM) (EC_50_ = 44 μM)	Chen et al. (2018), Zou et al. (2018), Rai et al. (2019), Wang et al. (2020), Wang et al. (2022), Yu et al. (2022)
Asari Radix et Rhizoma (ARR)	Rat	Agonist	Activates the channel	Methyl kakuo (EC_50_ = 0.27 μM) Amide A (EC_50_ = 0.47 μM) Asarinin (EC_50_ = 3.1 μM) Sesamin (EC_50_ = 2.3 μM)	Matsumoto et al. (2020), Jiang et al. (2021)
Notopterygii Radix et Rhizoma (NRR)	Mouse	Reduces cold and mechanical allodynia and AITC-induced pain	Downregulates expression and inhibits activation	not determined	Azietaku et al. (2017), Jiang et al. (2020), Ruan et al. (2023), Ye et al. (2023), Chen et al. (2024a)
Schisandrae Chinensis Fructus (SCF)	Guinea Pig	Attenuates the CS-induced increase in infiltration of pulmonary neutrophils and total inflammatory cells, as well as pulmonary MDA, TNF-α, and IL-8 levels	Downregulates expression	not determined	Zhong et al. (2015), Li et al. (2018), Yuan et al. (2018), Fu et al. (2023)
Zanthoxyli Pericarpium (ZP)	Rat	Decreases the concentrations of IL-4, IgE, GRP, and histamine	Upregulates protein expression and downregulates mRNA expression	not determined	Zhou et al. (2022), Qi et al. (2024)
Perillae Fructus/Perillae Folium (PFB)	Rat	Agonist	Activates the channel	Perillaldehyde (EC_50_ = 41.0 ± 7.5 μM) Perillaketone (EC_50_ = 19.7 ± 1.7 μM)	Bassoli et al. (2009), Bassoli et al. (2013), Cattaneo et al. (2017)
Borneol (BO)	Moue	Alleviates mechanical and thermal hyperalgesia	Inhibits activity	Borneol (IC_50_ = 0.20 ± 0.06 μM)	Takaishi et al. (2013), Zhou et al. (2016), Wang et al. (2017), Mei et al. (2023), Tian et al. (2023)
Paeoniae Radix (PR)	Rat	Reduces the expression of proinflammatory cytokines such as IL-6 and TNF-α, preserves the structure of the colonic tissue, and reduces the infiltration of inflammatory cells	Activates and inhibits under different conditions	Paeoniflorin (not determined)	Yan et al. (2018), Zhang and Wei (2020), Zhan et al. (2021), Zhang et al. (2021a), Zhang et al. (2022)
Sophorae Flavescentis Radix (SFR)	Mouse	Rescues defects in epidermal keratinization and lymphocyte infiltration, downregulates the mRNA expression of IL-1β, TNF-α, CXCR3, CXCL10, IL-6, IL-10, IL-17A, and IL-31	Downregulates mRNA expression	Oxymatrine (not determined)	Zhong et al. (2017), Chen et al. (2020), Li et al. (2020), Zhu et al. (2020), Zhang et al. (2021b), Sun et al. (2022a)
Rhei Radix et Rhizoma (RRR)	not determined	Causes minor connective tissue hyperplasia and fibroblasts, with occasional granulocyte presence and lymph cell infiltration in the liver tissue	Unclear	not determined	Xiang et al. (2020), Li et al. (2021b)
Tetrapanacis Medulla (TM)	Rat	Reduces the levels of IL-1β, IL-6, and TNF-α in the serum of the CCI rat model	Downregulates expression	Hederagenin (not determined)	Zhang et al. (2020a), Huang et al. (2023)
Puerariae Lobatae Radix (PLR)	Rat	Ameliorates mechanical allodynia and thermal hyperalgesia	Downregulates mRNA expression	Puerarin (not determined)	Xia et al. (2018), Ren and Qu (2023)
Glycyrrhizae Radix et Rhizoma (GRR)	Mouse	Prevents increases in the number of inflammatory cells such as neutrophils and macrophages and the levels of cytokines such as TNF-α, IL-6, and IL-1β	Downregulates mRNA and protein expression	Liquiritin (IC_50_ = 16.2 ± 1.1 μM)	Yang et al. (2017), Liu et al. (2020)
Lycii Fructus (LF)	Rat	Decreases serum malondialdehyde and IL-6 and colonic TNF-α levels	Downregulates protein expression	*Lycium barbarum* Polysaccharides (not determined)	Chen et al. (2022), Xiao et al. (2022), Teixeira et al. (2023)
Waldheimia glabra (Decne.) Regel (WR)	Rat	Agonist	Activates the channel	(+)-Ludartin (EC_50_ = 36.9 ± 5.7 μM) B-ring-homo-tonghaosu (EC_50_ = 40.4 ± 3.8 μM)	Giorgi et al. (2017)
Kalopanax Pictus Nakai (KPN)	Human	Agonist	Activates the channel	Methyl syringate (EC_50_ = 507.4 µM)	Son et al. (2012a), Son et al. (2012b), Park et al. (2022)
Platycodi Radix (PlaR)	Mouse	Suppresses or reduces the production and release of TNF-α and IL-1β	Downregulates and upregulates the expression of mRNA and protein, inhibits activity	Platycodins D3 (not determined)	Zhang et al. (2020b), Yang et al. (2023)
Croci Stigma (CS)	Mouse	Evokes a dose-dependent acute nociceptive response in mouse model	Activates the channel	Safranal (EC_50_ = 9 ± 0.2 μM)	(Li Puma et al., 2019; Pourbagher-Shahri and Forouzanfar, 2023)

**TABLE 2:** List of TCM formulas that regulate TRPA1.

Name of the formula	Species tested	Herbs included	Regulatory effects on TRPA1	References
Danggui Sini Decoction	Rat	*Cinnamomum cassia* Presl, *Paeonia lactiflora* Pallas *Tetrapanax papyriferus* Radix *Angelica sinensis*, *Glycyrrhiza uralensis* Fisch *Asarum heterotropoides* *Ziziphus jujuba* Mill	Downregulates expression of the protein, inhibits protein activity	(Zhang et al., 2019; Huo et al., 2020; Di et al., 2024)
Zhenxin Anshen Formula	Mouse	Os Draconis Concha Ostreae Rhizoma Drynariae Poria Herba Lophatheri	Downregulates mRNA and protein expression	Zhao et al. (2023)
Houpo-Mahuang Decoction	Mouse	*Magnolia officinalis* Rehder et E.H. Wilson *Ephedra intermedia* Schrenk et C.A. Mey *Gypsum fibrosum* *Prunus armeniaca* L *Zingiber officinale* Roscoe *Asarum heterotropoides* F. Schmidt *Schisandra chinensis* (Turcz.) Baill., *Pinellia ternata* (Thunb.) Makino *Triticum aestivum* L	Downregulates mRNA and protein expression	Sun et al. (2022b)
Yanghe Decoction	Rat	Radix Rehmanniae Praeparata Colla Cornus Cervi Cortex Cinnamomi Cassiae Semen Sinapis Herba Ephedra Sinica Ginger charcoal Radix Glycyrrhizae	Downregulates mRNA and protein expression	Gong et al. (2022)
Goshajinkigan (Jisheng Shenqi Pills)	Rat	Rehmanniae radix Achyranthis radix Corni fructus Moutan cortex Alismatics rhizome Dioscoreae rhizome Plantaginis semen Hoelen, processed Aconiti tuber, Cinnamomi cortex	Inhibits activity	(Kato et al., 2014; Mizuno et al., 2014)
Banhasasim-tang (Banxia Xiexin Decoction)	Mouse	*Pinellia ternata* (Thunb.) Makino (tuber), *Scutellaria baicalensis* Georgi (root) *Zingiber officinale* Roscoe (fresh) (rhizome) *Panax ginseng* C.A.Mey. (root) *Glycyrrhiza uralensis* Fisch. Ex DC. (rhizome) *Ziziphus jujuba* Mill. (fruit) *Coptis chinensis* Franch. (rhizome) *Zingiber officinale* Roscoe (dried) (rhizome)	Inhibits activity	Choi et al. (2023)
Qingpeng Ointment	Mouse	*Oxytropis falcata* Bunge Rheum lhasaense *Aconitum pendulum* Busch, *Terminalia chebula* Retz (stoned), *Terminalia billerica* (Gaertn.) Roxb *Phyllanthus emblica* Linn *Styrax tonkinensis* (Pierre) Craib ex Hartw *Tinospora sinensis* (Lour.) Merr Synthetic musk	Downregulates mRNA expression	Gong et al. (2019)
Daikenchuto(Da-Jian-Zhong-Tang)	Mouse	Zingiberis Rhizoma (Ginger) *Panax ginseng* (Ginseng; Ginseng radix) Zanthoxyli fructus (Japanese pepper), malt sugar	Upregulates protein expression and activates the channel	Hiraishi et al. (2018)
Sanse Powder	Rat	*Forsythia suspensa* *Glycyrrhiza uralensis* *Salvia miltiorrhiza* *Gentiana macrophylla* *Chaenomeles sinensis* *Strychnos nux-vomica* *Ligusticum striatum* Hort *Curcuma longa* *Paeonia lactiflora* Notopterygium root *Saposhnikovia divaricata*	Downregulates mRNA and protein expression	(Wu et al., 2020; Li et al., 2021a)
Maobushisaishinto (Mahuang Fuzi Xixin Decoction)	Rat	Ephedrae Herba Aconiti Radix Processa Asiasari Radix	Activates the channel	Matsumoto et al. (2020)
Shugan Decoction	Rat	White atractylodes rhizome, white peony root, dried old orange peel, ledebouriella root Radix bupleuri	Inhibits activity	Shi (2015)
Tokishakuyakusan (Danggui Shaoyao San)	Rat	Peony Root *Atractylodes lancea* Rhizome Alisma Tuber Poria Sclerotium Cnidium Rhizome Japanese Angelica Root	Activates the channel	Shimizu et al. (2022)
Wu-Tou Decoction	Mouse	Aconiti Radix Cocta Herba Ephedrae Paeoniae Radix Alba Astragali Radix Glycyrrhiza Radix Preparata	Downregulates protein expression and inhibits activity	Wang et al. (2015)
San’ao Decoction	Mice	Herba Ephedrae Semen Armeniacae amarum Radix Glycyrrhizae	Downregulates mRNA and protein expression and inhibits activity	(Wang et al., 2019b; Wang et al., 2021)
Chi-Huang Solution	Mouse	*Scutellaria baicalensis* Georgi *Sophora flavescens* Aiton *Paeonia lactiflora* Pall *Rehmannia glutinosa* (Gaertn.) DC *Lycium barbarum* L *Plantago asiatica* L *Dictamnus albus* L *Cnidium monnieri* (L.) Cusson *Bassia scoparia* (L.) A.J.Scott	Downregulates mRNA expression	Yang et al. (2022)

TCM is of great value and possesses the potential to serve as a resource for the development of new drugs. However, further investigations of TCM drugs acting on TRPA1 still need to be conducted since the existing studies are relatively superficial and limited in number. For example, some TCM components have dual-directional regulatory effects on TRPA1 function and expression, indicating that these compounds show activating or inhibitory effects depending on their concentration. Apart from this, some TCM ingredients display mild and long-lasting desensitization effects on TRPA1, whereas other TCM drugs exhibit antagonistic effects on the channel. Moreover, the species investigated in current research are predominantly limited to rodents and humans. Since the compounds discussed are mainly non-electrophilic, their primary mode of interaction with TRPA1 is non-covalent binding, occurring within a pocket formed by the TRP-like domain and the pre-S1, S4, and S5 helices of one subunit (Gawalska et al., 2022). This pocket is species-specific, and therefore, the modulation of TRPA1 can differ across species, even for the same compound (Chen J. et al., 2013; Memon et al., 2019). Thus, studies involving more species are required to further explore the mechanisms and regulatory principles underlying TRPA1 modulation by TCM. All these special bioactivities differentiate them from the agonists, antagonists, and desensitizers that are already known, and may be the key point to clarify the efficacy of TCM. Therefore, the mechanisms underlying these effects require elucidation.

In conclusion, we hope that this review will provide reference for future TCM studies on TRPA1, facilitate the development of novel drugs, and offer new directions for modernization of TCM.
